# Memristive Baffle Systems: Design, Simulation, and Applications

**DOI:** 10.1002/advs.202523273

**Published:** 2026-03-03

**Authors:** Eun Young Kim, Juseong Park, Sumin Ju, Taeyoung Jeong, Dohyun Kim, Woojoon Park, Myeongchan Ko, Woon Hyung Cheong, Jung‐Hae Choi, Kyung Min Kim

**Affiliations:** ^1^ Graduate School of Semiconductor Technology Korea Advanced Institute of Science and Technology (KAIST) Daejeon Republic of Korea; ^2^ Department of Materials Science and Engineering Korea Advanced Institute of Science and Technology (KAIST) Daejeon Republic of Korea; ^3^ Electronic and Hybrid Materials Research Center Korea Institute of Science and Technology (KIST) Seoul Republic of Korea; ^4^ Department of Materials Science and Engineering and Inter‐University Semiconductor Research Center Seoul National University Seoul Republic of Korea

**Keywords:** baffle system, COMSOL multiphysics, DFT, endurance, HfO_2_, memristor, synaptic characteristic, valence change memory

## Abstract

Valence change memory (VCM)‐based memristors have emerged as promising artificial synapses for neuromorphic computing, yet their nonlinear conductance modulation and limited endurance remain major bottlenecks. Here, we introduce the baffle system concept into HfO_2_‐based VCM memristors to regulate oxygen vacancy (V_O_) transport and improve both synaptic linearity and cycling reliability. Guided by a multiscale simulation framework that integrates density functional theory calculations and finite element method‐based multiphysics modeling, dual Al_2_O_3_ interlayers were strategically incorporated as nanoscale barriers within the HfO_2_ matrix to modulate V_O_ migration. The appropriately engineered baffle barriers promote lateral filament growth, enhancing synaptic linearity by up to 43%, while simultaneously confining V_O_s to achieve over a 60× improvement in endurance. The proposed memristive baffle system provides a practical materials design strategy for precise control of ion transport and filament dynamics, contributing to the development of advanced memristive materials and enhancing their feasibility for neuromorphic applications.

## Graphical abstract

This study presents a nanoscale baffle‐system strategy for HfO_2_‐based memristors that controls oxygen‐vacancy transport through Al_2_O_3_ interlayers. Guided by multiscale simulation and experimental validation, the Al_2_O_3_ dual‐barrier configuration simultaneously enhances synaptic linearity and endurance. The baffle concept provides a materials‐design approach for reliable and energy‐efficient memristive devices applicable to neuromorphic computing systems.

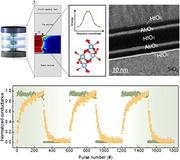

## 1 Introduction

**FIGURE 1:**
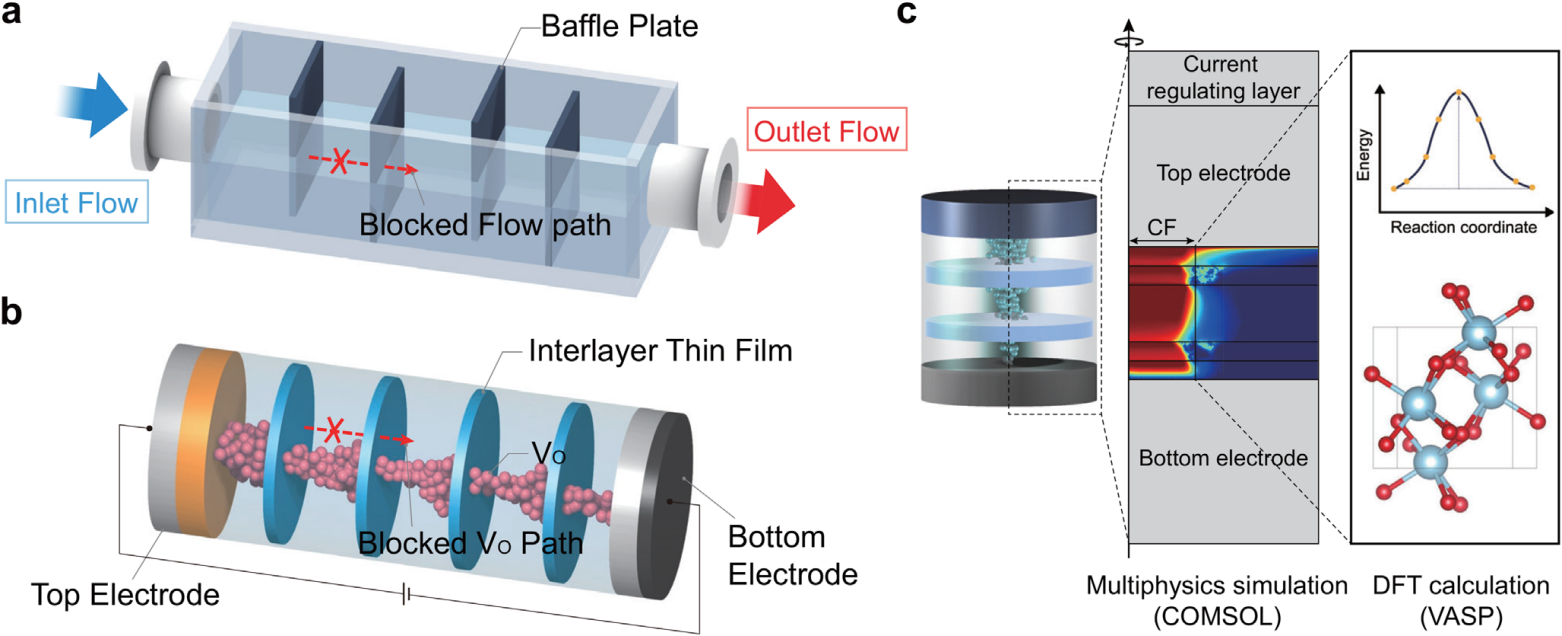
Schematic representation of nanoscale baffle systems and key concepts of multiscale simulation. (a) A baffle system used for physical control over heat, wave, or fluid flow. The inlet and outlet regulate directional flow, creating a controlled environment for various industrial processes. (b) A memristive baffle system enabling both physical and chemical control over ion movement and redox reactions within the device structure. (c) Multiscale simulation combining multiphysics‐based finite element modeling and DFT calculations to design the memristive baffle system of VCM memristors.

Baffle systems are widely utilized in macroscopic dynamics to regulate the flow of fluids or gases [1], enhance heat transfer efficiency [2], and control the rates of chemical reactions [3] (Figure 1a). These systems employ physical barriers or structural elements, such as inclined plates, to modify the flow paths or velocities of fluids, for improving system stability and yielding additional functional benefits. Although the baffle systems are extensively applied in macroscopic contexts, their application in nanoelectronics—specifically for the precise control of electron and ion transport—remains largely unexplored.

In this context, it is worth considering the application of baffle systems in memristive devices, especially in valence change memory (VCM) memristors (Figure 1b). They have attracted significant attention for artificial synapse elements in neuromorphic systems with advantages such as their CMOS compatibility [4], high reliability [5], analog switching capabilities [6], electroforming‐free operation [7], and multimodal functionalities [8]. Resistive switching in filamentary VCM devices is most commonly attributed to the electric field‐driven migration of oxygen vacancies (V_O_s), which modulates the interfacial barrier and the continuity of conductive filaments (CFs) [9]. Although various ionic defects may exist in HfO_2_‐based oxides, V_O_s are widely regarded as the dominant mobile species due to their comparatively low migration barriers and faster kinetic response under an applied electric field [10].

However, it should be noted that this V_O_‐based description represents a dominant but not exclusive switching mechanism. First‐principles studies have predicted that cationic interstitials can also act as positively charged donor‐type defects and potentially participate in filament formation, depending on material composition, stoichiometry, and local chemical environments [10, 11, 12]. These alternative ionic defects have recently been highlighted as potentially overlooked contributors in memristive systems. Therefore, for a more comprehensive and highly accurate simulation, it may be ideal to account for the transport of all potential mobile defect contributors. Nevertheless, we treated only V_O_s as the primary mobile species governing the filamentary switching in HfO_2_‐based VCM devices, as our proposed simulation framework is designed as an advanced extension of the conventional V_O_‐based simulation methodologies.

Building on this V_O_‐dominated switching mechanism, previous studies have demonstrated that interfacial and interlayer engineering can effectively regulate V_O_ transport pathways and the spatial evolution of CFs. The insertion of secondary oxide layers has been reported to modulate vacancy flux, confine filament growth, and improve switching stability and analog conductance behavior [13, 14, 15]. These observations indicate that V_O_ migration is not merely a localized interfacial phenomenon, but rather a spatially distributed transport process within the switching layer.

From this perspective, the collective movement of V_O_s can be interpreted within the framework of continuum mechanics, where electric field‐driven drift and concentration gradient‐induced diffusion dynamics interact. Therefore, they can be interpreted as a fluid flow, indicating the potential applicability of baffle systems for actively regulating these dynamics.

The baffle system is commonly used to control and stabilize fluid flow. This concept can be applied to a memristive baffle system, offering a potential solution to improve the current limitations of VCM devices. In this context, we focus on two key characteristics related to these functions: synaptic linearity, which is associated with fluid flow control, and endurance, which is linked to flow stability.

First, in the application of VCM memristors as artificial synapses, linearity in potentiation and depression (P/D) characteristics is critical because it ensures consistent synaptic weight updates during neural network learning, regardless of the current conductance state [16]. However, conventional VCM memristors have struggled to achieve good linearity due to the highly nonlinear dependence of V_O_ migration on the electric field. Although several studies have attempted to improve synaptic linearity, they have primarily relied on external methods, such as narrowing the operating window [17] or continuously modulating the input pulse shape [18]. Although these approaches are effective, a more fundamental solution that adjusts the intrinsic material properties to improve synaptic linearity would offer greater opportunities for the practical utilization of synaptic devices, which could be feasible through the application of baffle system concepts.

Second, the excellent endurance characteristics of VCM memristors have been widely reported [19, 20, 21, 22, 23, 24, 25, 26, 27], and these are generally attributed to the stable retention of V_O_s within the switching layer, which move collectively back and forth during switching. Therefore, a stable material structure that prevents V_O_ depletion is essential, and this can be interpreted and approached as achieving flow stability of the baffle system. This perspective provides a new physical framework for analyzing the stability and reliability of memristive devices and offers valuable insights into improving them.

In this study, we propose a multilayered VCM memristor structure incorporating the baffle system concept, thereby effectively improving synaptic linearity and endurance. To successfully develop the memristive baffle system, we first established a material design methodology based on multiscale simulations that combines Density Functional Theory (DFT) calculations with multiphysics‐based modeling to ensure the reliability of the simulation results (Figure 1c). Using this framework, we simulated various material parameters and insertion locations for the baffle barrier in a HfO_2_‐based VCM memristor and evaluated their effects on synaptic linearity and endurance. The results suggested that introducing double Al_2_O_3_ interlayers (ILs) as baffle barriers could simultaneously enhance both properties, which we subsequently validated through experimental demonstration. These results demonstrate the feasibility of applying baffle systems to nanoelectronics and pave the way for new strategies in materials design.

## 2 Results and Discussion

### 2.1 Multiscale Simulation of HfO_2_‐Based VCM Device

**FIGURE 2:**
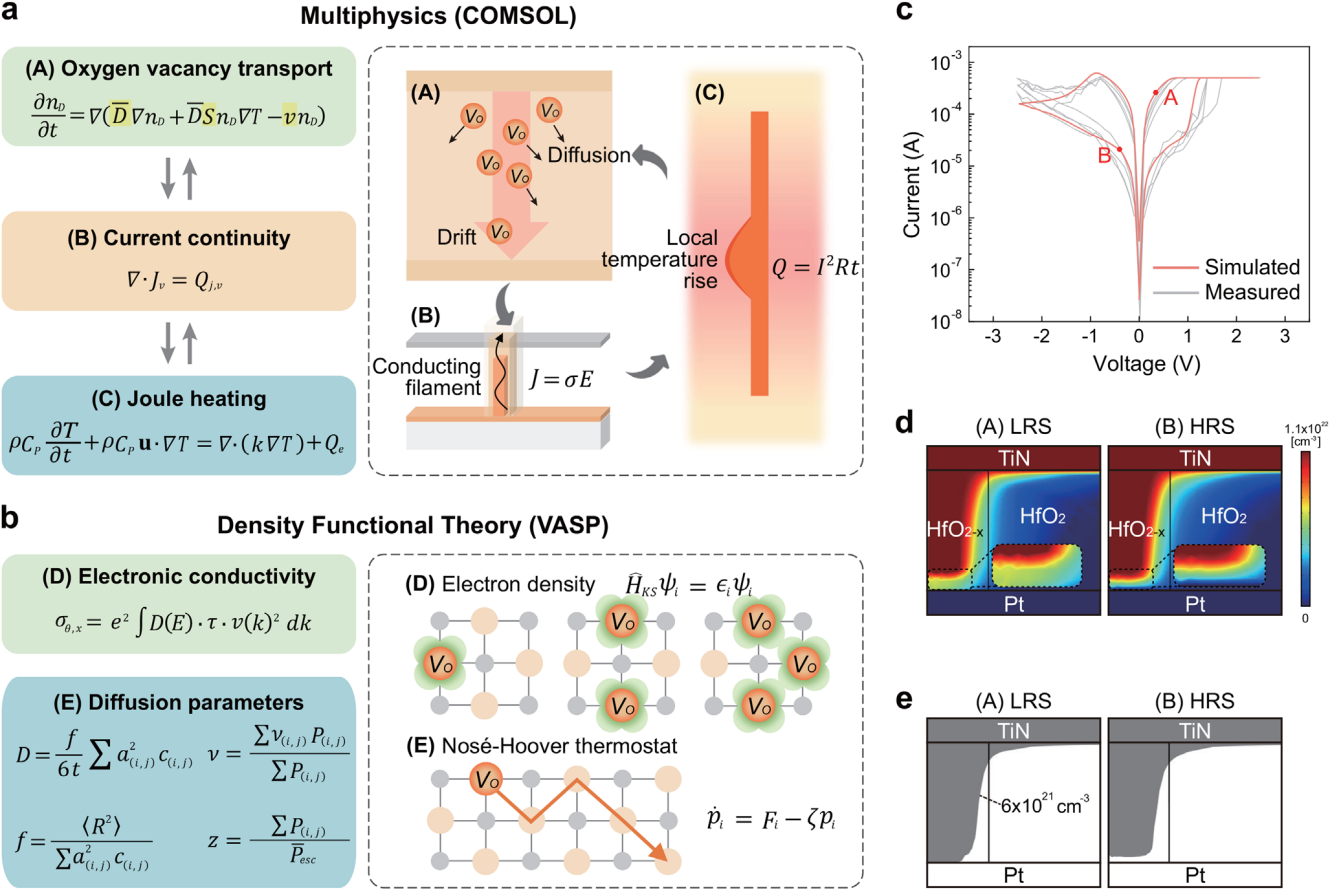
Multiscale simulation frameworks and validation for a single HfO_2_ VCM device. (a) Overview of the COMSOL Multiphysics simulation framework, solving oxygen vacancy transport, current continuity, and Joule heating dynamics. Here, the key material parameters (yellow) were obtained from the DFT calculation. (b) Overview of the DFT calculation framework used to extract key parameters from electron‐density and molecular‐dynamics analyses for input into the COMSOL model. (c) Simulated and experimental *I‐V* characteristics of the single HfO_2_ device. (d) Simulated V_O_ distributions for the single HfO_2_ device at the LRS (point A) and HRS (point B) in (c). (e) The filament configuration at the LRS and HRS, illustrating filament formation and rupture near the bottom electrode.

Multiphysics‐based modeling simplifies the complex motion of ions by approximating it as collective fluid dynamics, thereby significantly deepening our understanding of the VCM mechanisms [27, 28, 29, 30, 31, 32]. Figure 2a presents the multiphysics‐based modeling framework implemented in COMSOL Multiphysics, illustrating the three coupled physical dynamics (V_O_ transport, current continuity, and Joule heating). Here, we assumed that V_O_s are the primary mobile species governing the filamentary switching in HfO_2_‐based VCM devices, consistent with prior multiphysics‐based modeling works [27, 28, 29, 30]. These governing equations are used to simulate V_O_ movement under the combined influence of electric field and temperature during memristive operation, where V_O_s are treated as a representative mobile ionic species to capture generic drift‐diffusion characteristics in HfO_2_‐based VCM devices. Also, while the local temperature rise due to Joule heating during switching is included in our simulation, we used material parameters at room temperature and assumed they remain constant with respect to temperature. Although this simplification does not fully capture temperature‐dependent property variations within the heated filament region, it is a commonly adopted assumption in prior modeling studies and does not significantly affect the overall switching characteristics. Detailed multiscale simulation conditions for the VCM model are illustrated in Figure S1 and Table S1 [27, 28].

One crucial consideration in this simulation approach is that there can be multiple parameter combinations capable of reproducing the experimental results. In other words, multiple sets of parameters can produce the same current–voltage (*I*–*V*) characteristics. Therefore, when selecting material parameters for reproducing a specific *I*–*V* curve, it is necessary to provide a clear and reasonable explanation of how these parameters were determined. Previous studies have primarily relied on literature‐reported values to justify their choice, and this approach has been widely accepted. However, its appropriateness remains controversial. In particular, for this method to be extended beyond fitting existing data to predicting the behavior of new devices, it is necessary to adopt more suitable material parameters.

To quantitatively bridge atomistic and continuum scales, Density Functional Theory (DFT) calculations were conducted, as illustrated in Figure 2b. The DFT framework was used to evaluate the electronic conductivity and the diffusion parameters of V_O_s in HfO_2_, which serve as key inputs for the COMSOL Multiphysics model. Specifically, we extracted the hopping distance (*a*), activation energy for migration (*E_a_*), pre‐exponential conductivity factor (*σ_0_*), correlation factor (*f*), and jump‐attempt frequency (*ν*) from the electron‐density and molecular‐dynamics analyses based on the Nosé−Hoover thermostat. Among these, even small variations in *a*, *E_a_*, or *σ_0_* were found to critically influence the *I*–*V* characteristics, while *f* and *ν* together determined the overall diffusion coefficient and temperature dependence of vacancy mobility. More details of these DFT calculations are presented in Figures S2 and S3, Table S2, and reference [33].

Figure 2c compares the *I*–*V* characteristics obtained from the multiscale simulation (red lines) with the experimental *I*–*V* characteristics (gray lines) of the HfO_2_ VCM device (TiN(40 nm)/HfO_2_(14 nm)/Pt(40 nm) stack), confirming the consistency and validity of the simulation. Figure 2d shows the simulated V_O_ distributions at a low resistance state (LRS, marked as A in Figure 2c) and a high resistance state (HRS, marked as B in Figure 2c). The inset enlarges the bottom interface region to show the V_O_ distributions at the switching region clearly. Figure 2e represents the contour line corresponding to a V_O_ concentration of 6 × 10^21^ cm^−3^ [34], which delineates the boundary between the CF and insulating regions. These results clearly demonstrate the localized interfacial switching characteristics typically observed in conventional VCM devices [35]. In addition, the residual filament exhibits a conical shape, which is also consistent with the established VCM theory [36].

### 2.2 VCM Device Simulation with V_O_ Baffle System

#### 2.2.1 Enhancing Synaptic Characteristics by V_O_ Drift Control

The distinctive feature of our simulation methodology is that our model not only reproduces existing results but also accurately predicts the behavior of newly designed structures, which is a capability that has not been achieved in prior simulation studies. To validate this capability, we designed a memristive baffle system and applied it to enhance the synaptic linearity characteristics of the VCM memristors. The proposed system is a multilayered structure in which the IL serving as a baffle barrier is inserted into a conventional single‐layer HfO_2_ memristor. Using multiscale VCM simulations, we examined whether inserting an IL with physical properties distinct from those of HfO_2_ could enhance synaptic performance. For this purpose, we systematically varied three key physical parameters of the IL—hopping distance (*a_IL_*), activation energy for V_O_ migration (*E_a, IL_*), and pre‐exponential factor of electrical conductivity (*σ_0, IL_* ​)—and evaluated their influences on the synaptic linearity characteristics.

**FIGURE 3:**
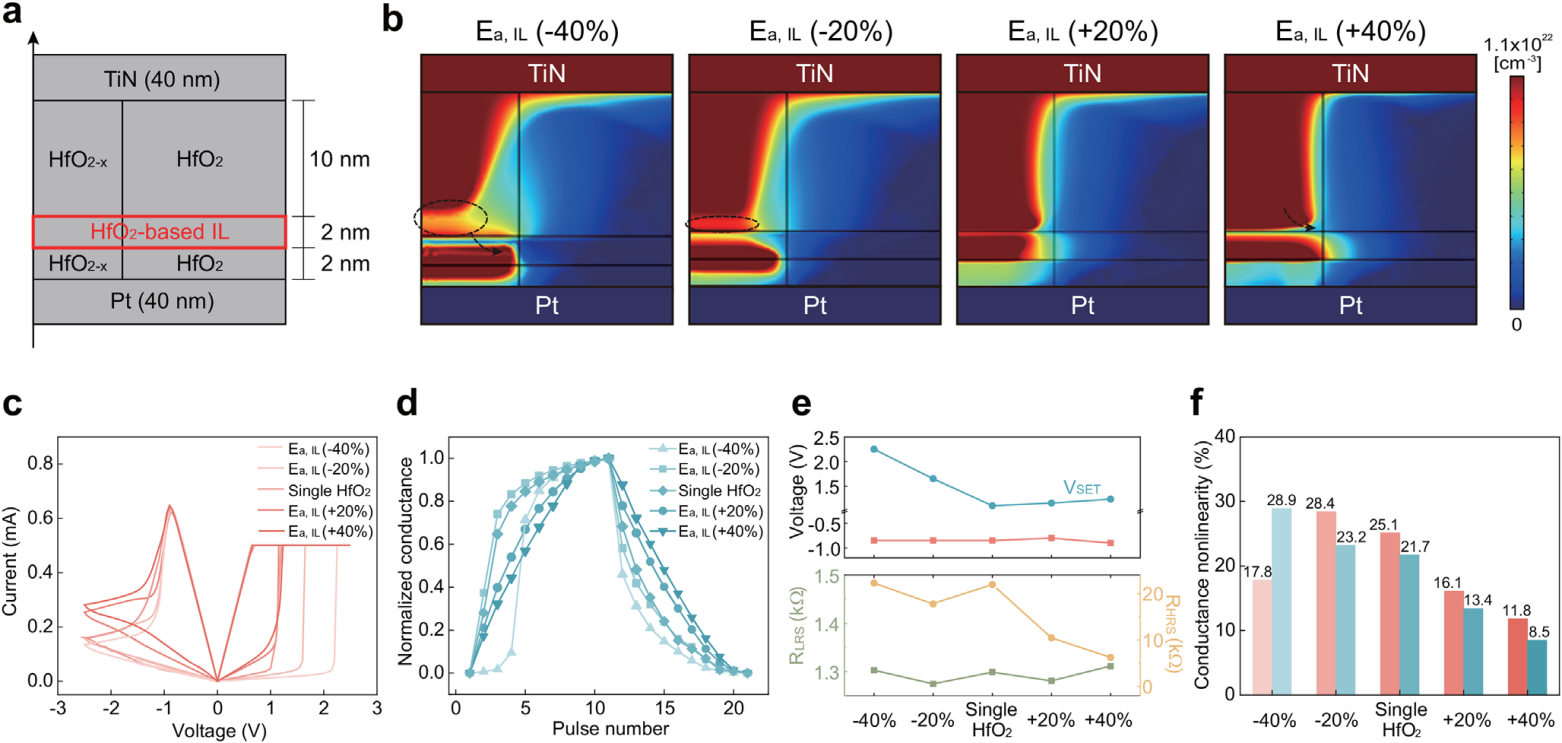
Simulation results of VCM‐based memristive baffle devices. (a) Cross‐sectional schematic of the simulated device structure, including a 2 nm‐thick IL placed 2 nm above the bottom electrode. (b) Simulated V_O_ distributions at the LRS for various *E_a, IL_*, ranging from −40% to +40% relative to HfO_2_. (c, d) Corresponding changes in (c) *I‐V* curves and (d) P/D characteristics for different *E_a, IL_* conditions. (e) *V*_SET_ and *V*_RESET_ (upper panel), and *R*_LRS_ and *R*_HRS_ (lower panel) obtained from the *I‐V* curves. (f) *NL* values obtained from the P/D characteristics, demonstrating improved synaptic linearity with increasing *E_a, IL_*.

Among the possible IL configurations of location and thickness, we selected the structure shown in Figure 3a, where a 2 nm‐thick IL is inserted near the bottom interface (2 nm from the bottom). This region is where CF formation and rupture primarily occur; therefore, inserting the IL at this location exerts the greatest influence on synaptic behaviors. Also, among the three key parameters, we discuss representative results for varying *E_a_*, which ultimately had the most meaningful impact on synaptic linearity characteristics (the simulation results for the other key parameters are provided in Figure S4).

Figure 3b presents the simulation results of V_O_ distributions in the LRS after SET switching, in which the *E_a_* of the IL (*E_a, IL_*) were set to 0.42 eV (−40% of 0.70 eV of *E_a_*_, HfO2_), 0.56 eV (−20%), 0.84 eV (+20%), and 0.98 eV (+40%) to investigate a clear trend of the *E_a_* modulation. More simulation results for different *a_IL_* and *σ_0,IL_* of IL can be found in Figure S4. The V_O_ distributions in the LRS were evaluated because this state represents the fully developed CF, where the influence of the IL barrier on V_O_ localization can be most clearly observed. When the *E_a, IL_* was lower than *E_a_*_, HfO2_, the baffle barrier facilitated the migration of V_O_s, thereby accelerating the SET process. In contrast, when the *E_a, IL_* was higher than *E_a_*_, HfO2_, the V_O_s tended to stay longer within the IL, resulting in reduced migration toward the bottom electrode (BE) as *E_a, IL_* increased.

This influence of the IL is directly reflected in both the *I–V* (Figure 3c) and P/D characteristics (Figure 3d). Figure 3e,f summarize switching parameters (*V_SET_*, *V_RESET_*, *R_LRS_*, and *R_HRS_*) from the *I*–*V* characteristics and the synaptic linearity from the P/D characteristics for various *E_a, IL_* values, respectively. Here, the non‐linearity (*NL*) was calculated using the following equation [37]:

(1) $$NL=\left|\frac{G_{mid}-G_{s}}{G_{mid}}\right|\;\times 100\%$$

where *G_mid_* is the midrange of the maximum and minimum conductance ((*G_max_* + *G_min_*) /2), and *G_s_* ​ refers to the conductance at the mid‐point of the full P/D curves.

In the HRS and during SET switching, as shown in Figure 3e, *V*_SET_ and *R*_HRS_ tend to increase as *E_a_* decreases. These results can be attributed to rapid V_O_s migration within the IL, leading to their accumulation at the IL/HfO_2_ interface. This accumulation induces an additional gap in the vertical V_O_ distribution in the residual filament (black dashed circle in Figure 3b), resulting in the higher *V*_SET_ and *R*_HRS_. Additionally, filament formation occurs abruptly at higher voltages, which adversely affects the synaptic characteristics (Figure 3f). When *E_a_* increases, *V*_SET_ and *R*_HRS_ show little variation, which is reasonable since the IL no longer induces additional gaps along the vertical direction. Meanwhile, the *NL* characteristics improve gradually because the IL promotes horizontal filament growth, making the CF more cylindrical and leading to a gradual decrease in resistance. In summary, an IL with a higher *E_a_* facilitates lateral CF expansion rather than vertical formation, thereby improving the NL. This result provides a guideline for designing the properties of the IL, and the experimental validation is presented in the following section.

In the LRS and during RESET switching, *V*_RESET_ and *R*_LRS_ remain nearly constant regardless of *E_a_*, since the IL has little influence on the CF rupture processes occurring near the interface. However, the NL improves as *E_a_* increases, consistent with the underlying principle observed in the SET process.

#### 2.2.2 Enhancing Endurance via V_O_ Isolation

Next, we investigated the impact of IL insertion on endurance. Endurance testing requires numerous repeated SET and RESET switching cycles; however, running even a single cycle in simulation is time‐consuming, making it difficult to fully implement endurance cycle testing through simulation alone. Therefore, we aimed to predict endurance characteristics by correlating the endurance degradation mechanisms with the V_O_ distributions obtained from simulation.

In VCM memristors, endurance degradation is generally attributed to the loss of V_O_s within the film, primarily due to two factors: oxidation (or oxygen incorporation) through the electrodes and the formation of sub‐oxide phases [38]. In the HfO_2_‐based VCM memristor, since the HfO_2_ does not form sub‐oxide phases, V_O_ loss mainly occurs through oxidation. Specifically, this process is expected to take place at the top electrode (TE) interface, as oxidation is thermodynamically driven and therefore closely related to the local concentration of reactants. At the TE interface, the reactant concentrations—V_O_s in the residual filament and oxygens supplied from the electrode—are the highest, resulting in a greater likelihood of oxidation compared to the BE interface or the bulk region.

Therefore, the degree of endurance degradation can be predicted by comparing the V_O_ concentration at the TE interface. Based on this understanding, we performed multiple SET and RESET switching cycles in the simulation, varying the baffle barrier configuration (i.e., the thickness and location of the IL and their key parameters), as in the previous synaptic characteristics simulation, and analyzed the resulting changes in V_O_ concentration and spatial distribution. Among the different configurations, we compare two representative cases here: one with the IL placed near the upper side (2 nm below the TE) and the other with the IL near the bottom side (2 nm above the BE). For the IL, its thickness was fixed at 2 nm, and a high *E_a, IL_* condition was adopted, which was empirically concluded to be the most effective after extensive simulations.

**FIGURE 4:**
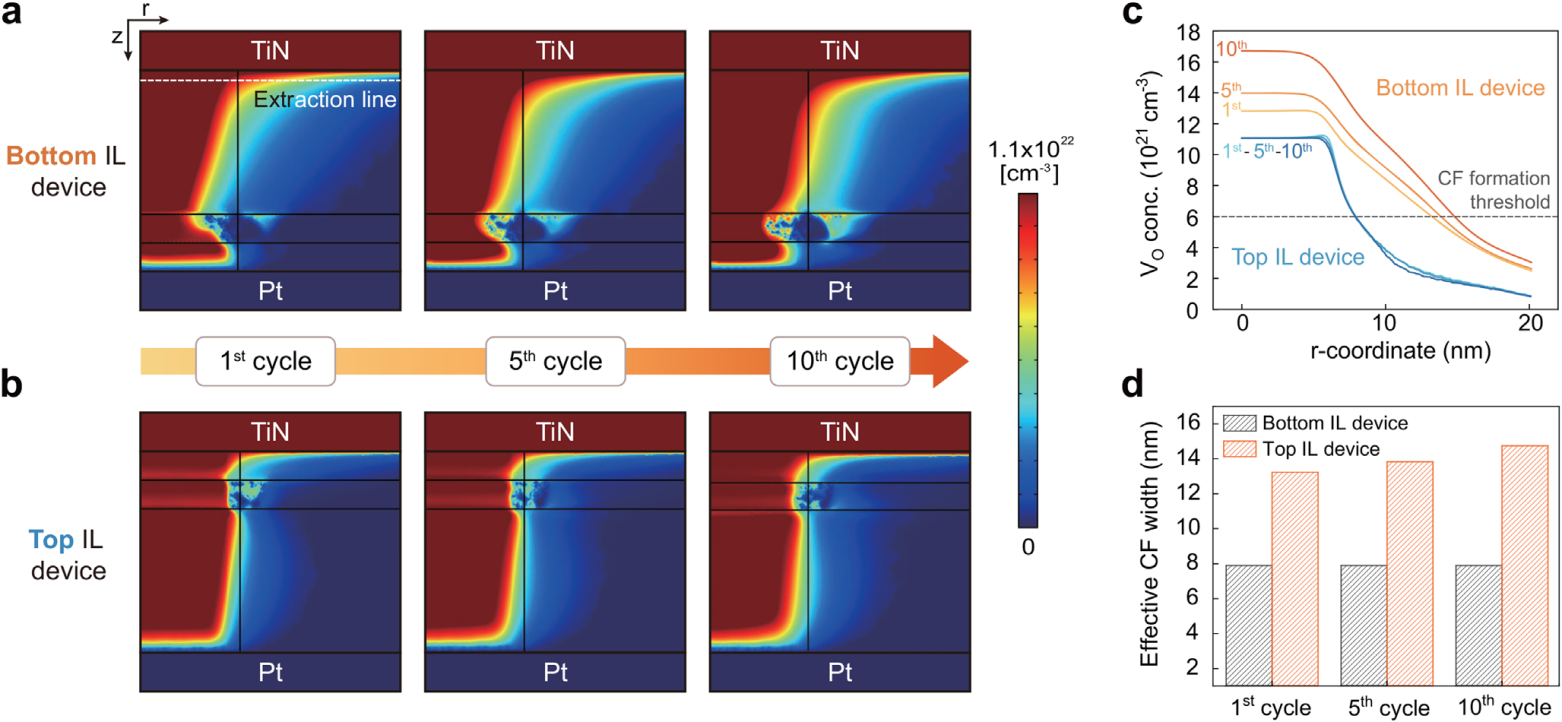
Simulation results showing the change in V_O_ distribution with repeated switching cycles. (a, b) Simulated V_O_ concentration profiles after the first, fifth_,_ and 10th SET and RESET cycles for (a) the bottom IL device and (b) the top IL device, respectively. (c) A horizontal line profile of V_O_ concentration near the TE (0.5 nm below the TE interface) as the switching cycle repeats, comparing the bottom and top IL devices. (d) CF width change at the first, fifth, and 10th cycles, where CF is defined based on a CF formation threshold of 6 × 10^21^ cm^−3^.

Figure 4a,b shows the simulated V_O_ distributions in the HRS with the IL located near the bottom and top interfaces, respectively, after first, fifth, and 10th SET and RESET cycles. To quantitatively compare the two cases, Figure 4c shows the V_O_ concentration profile along the horizontal line (indicated by a dashed line) located 0.5 nm below the TE interface, where vacancy accumulation predominantly occurs. Here, the region with V_O_ concentration higher than 6 × 10^21^ cm^−3^ can be considered as the effective filament. The single bottom IL device exhibits a continuous increase in vacancy density and filament width, suggesting that it cannot fully suppress vacancy migration toward the opposite electrode, and oxidation may accelerate with cycling, whereas the top IL maintains the V_O_ concentration and the filament width over multiple switching cycles. This is because the back‐and‐forth movement of V_O_s is restricted to the lower region of the IL due to its high *E_a_*. Overall, assuming that endurance degradation mainly originates from oxidation at the TE interface, placing the IL near the top interface provides better control of V_O_ distribution and improves cycling stability.

### 2.3 Dual Al_2_O_3_‐Inserted HfO_2_ Memristor

#### 2.3.1 Synaptic Performance of the Al_2_O_3_ Dual‐Barrier Device

**FIGURE 5:**
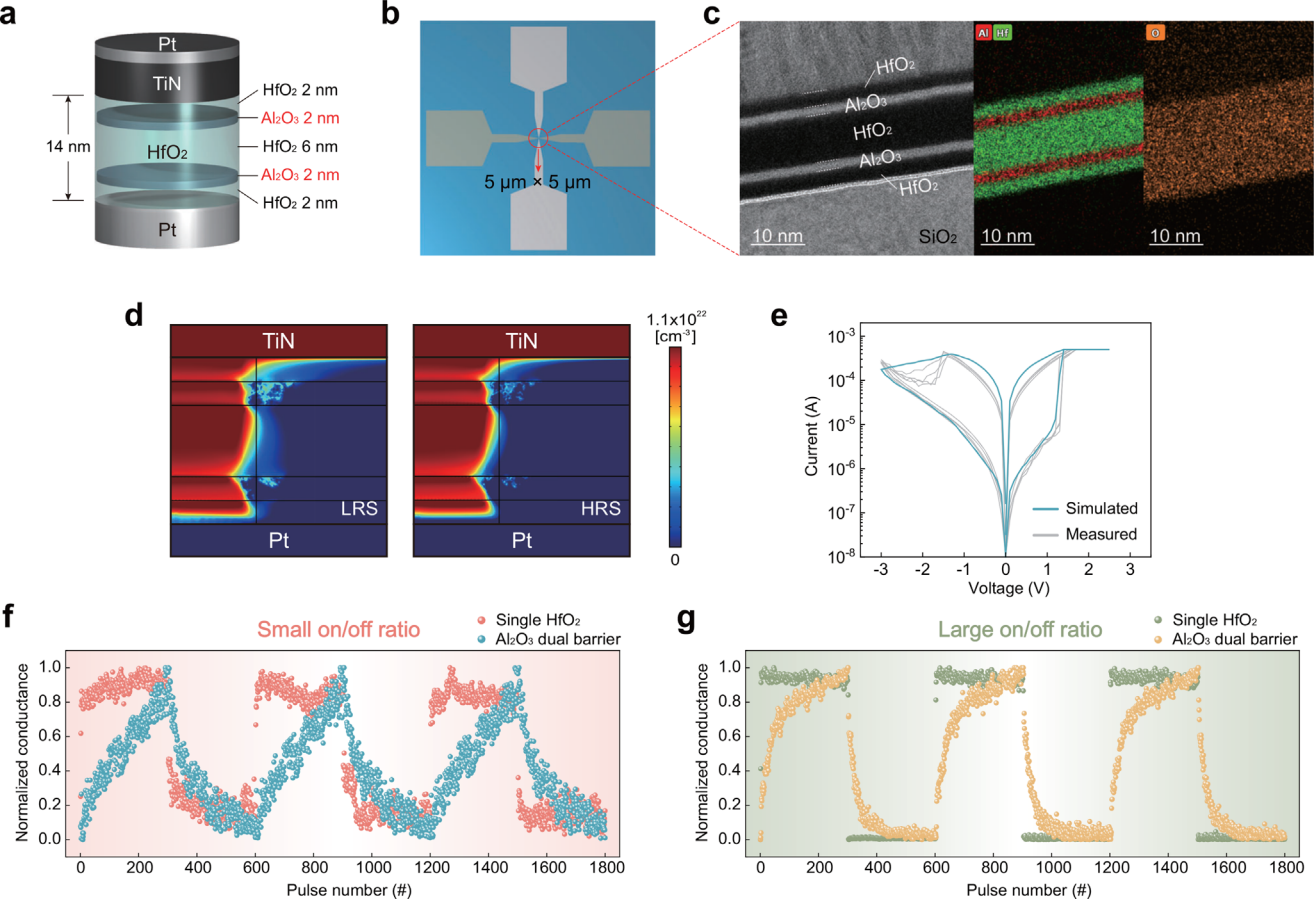
Experiment and simulation results of the Al_2_O_3_ dual‐barrier device. (a) Schematic structure of the Al_2_O_3_ dual‐barrier device. (b) OM image and (c) cross‐sectional TEM image and corresponding EDS results for Al, Hf, and O of the dual‐barrier device. (d) V_O_ distributions at LRS (left) and HRS (right). (e) Comparison of simulated and experimental *I‐V* characteristics. (f, g) Comparison of P/D characteristics of the single HfO_2_ device and the dual‐barrier device operating in (f) a small‐window mode and (g) a large‐window mode.

Based on the previous simulation results suggesting that the bottom IL with a higher E_a_ enhances synaptic linearity while the top IL with a higher E_a_ improves endurance, we selected Al_2_O_3_ as the IL material because its E_a_ is 0.9 eV [39], which is higher than 0.7 eV in HfO_2_. In addition, Al_2_O_3_ is readily deposited with precise thickness control by atomic layer deposition (ALD), forms a stable interface with HfO_2_ without introducing undesirable interfacial states, and is highly compatible with filamentary VCM devices. Several candidate interlayer materials were considered, and the reasons for their exclusion are summarized through comparative analyses in the Supplementary Information (Figures S5 and S6 and Table S4). The Al_2_O_3_ dual‐barrier device was then fabricated with a multilayer structure of HfO_2_ (2 nm)/Al_2_O_3_ (2 nm)/HfO_2_ (6 nm)/Al_2_O_3_ (2 nm)/HfO_2_ (2 nm) (Figure 5a), and simulations were performed for this configuration. Figure 5b shows a top‐view optical microscope (OM) image of the device with the cell dimensions of 5 µm × 5 µm in a crossbar structure. Figure 5c presents the cross‐sectional transmission electron microscopy (TEM) image and energy dispersive spectroscopy (EDS) mapping images of Al (red), Hf (green), and O (orange), confirming the device stack.

Figure 5d shows the simulation results of the LRS and HRS (a more detailed initial configuration of the model setup is described in Figure S7). The filament width within the Al_2_O_3_ layers was narrower than that in the surrounding HfO_2_ regions, indicating that the Al_2_O_3_ layers locally suppress V_O_ migration within the bulk HfO_2_ and serve as active barriers that regulate filament growth. More discussion on the Al_2_O_3_ dual‐barrier device simulation results can be found in Figure S7. Figure 5e compares the measured and simulated I‐V characteristics of the Al_2_O_3_ dual‐barrier device, confirming that the device's behavior closely matches the predictions of the simulation.

Next, we evaluated the P/D characteristics of the dual‐barrier device. Here, in general, the *NL* and the operating on/off window, which determines the multilevel capability of the synaptic states, exhibit a trade‐off relationship. For example, utilizing a smaller on/off window can improve the *NL* but limits the implementation of multilevel conductance states in the P/D operation. Therefore, both factors must be carefully balanced. To this end, we compared the P/D characteristics under two operating modes: a small‐window mode and a large‐window mode. Figure 5f shows the small‐window P/D characteristics with a weaker stimulation (+1.8 V for potentiation and −1.8 V for depression for 1 ms). With the baffle barriers, the *NL* values were improved from *NL*_pot_ = 19% and *NL*_dep_ = 13% with R_on/off_ = 1.3 (red dots) to *NL*_pot_ = 5%, and *NL*_dep_ = 6% with R_on/off_ = 1.4 (blue dots). Figure 5g shows the large‐window P/D characteristics with stronger stimulation (+2.5 V for potentiation and −2.5 V for depression for 1 ms). In this case, the *NL* values were changed from *NL*_pot_ = 81% and *NL*_dep_ = 84% with R_on/off_ = 13.5 (green dots) to *NL*_pot_ = 38%, and *NL*_dep_ = 42% with R_on/off_ = 52 (yellow dots). In both modes, the Al_2_O_3_ dual‐barrier device exhibited significantly improved linearity compared to the reference devices.

This improvement in synaptic linearity can be interpreted from the perspective of electronic transport at the electrode/oxide interface. At the Pt/HfO_2_ interface, a Schottky‐type potential barrier governs carrier injection in the low‐conductance regime, and its height and width are dynamically modulated by V_O_ accumulation under bias [40]. In the proposed dual‐barrier device, the Al_2_O_3_ interlayers limit the V_O_ flux reaching the interface, thereby suppressing abrupt barrier modulation and enabling gradual conductance updates with improved linearity [41].

Table 1 summarizes reported HfO_2_‐based synaptic devices designed to improve performance through structural modification. The proposed Al_2_O_3_ dual‐barrier device demonstrates significant linearity improvements, even under large conductance windows, offering a substantial advancement toward the practical implementation of artificial synaptic devices [42].

**TABLE 1:** Comparison of on/off ratio and linearity between the proposed device and various devices with modified HfO_2_‐based resistive switching layer structures.

Device structure	R_ON_/R_OFF_	# of states (pulse number)	Conductance window	Refs.
Pt/HfO_2_ nanorods/TiN	250	50	1.48	[43]
Ti/TiO_x_/HfO_2_/Pt	40	100	17.00	[44]
TiN/HfO_2_/WS_2_/Pt	25	50	1.09	[45]
Ag/HfO_2_/SiO_2_/Pt	20	100	1.20	[46]
Ni/TaN/nanocrystal‐HfO_2_/ITO	80	100	1.47	[47]
TaN/Ni/HfO_2_/Al_2_O_3_/HfO_2_/ITO	70	50	3.00	[15]
TiN/Ti/HfO_x_/AlO_x_/TiN	200	64	2.25	[48]
Pt/HfO_x_/AlO_x_/TaN	400	100	1.43	[14]
Ti/HfO_x_/AlO_y_ superlattice layer/TiN	30	100	5.50	[49]
Pt/AlO_x_/HfO_x_/TiN	25	180	1.56	[50]
**TiN/HfO_2_/Al_2_O_3_/HfO_2_/Al_2_O_3_/HfO_2_/Pt**	**60**	**300**	**1.4**	**This work**
		**300**	**52**	

#### 2.3.2 Switching Speed and Endurance of the Devices

**FIGURE 6:**
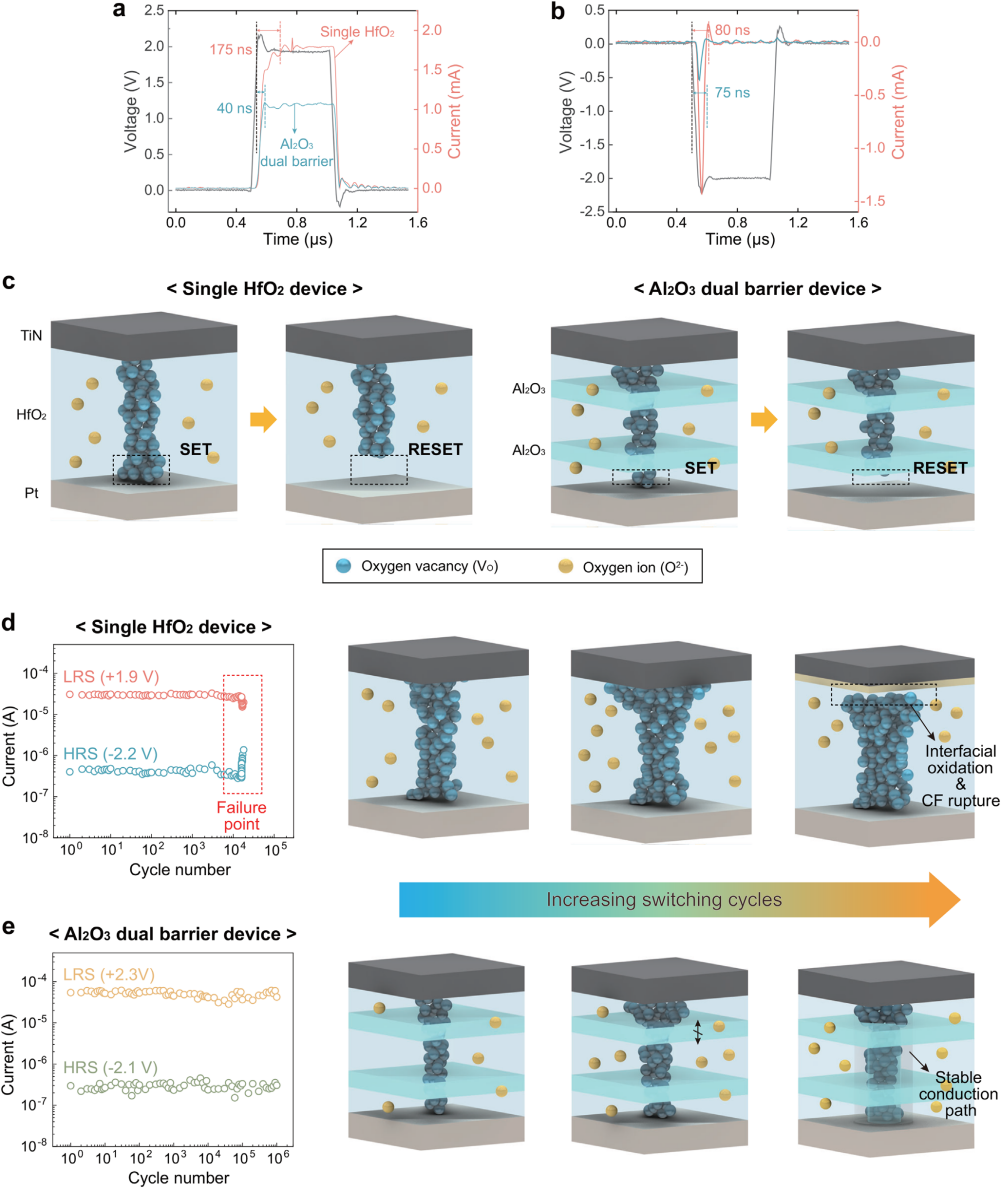
Comparison of switching dynamics and endurance characteristics between single HfO_2_ and Al_2_O_3_ dual‐barrier devices. (a, b) Experimentally measured transient current responses at (a) SET and (b) RESET switching in the single HfO_2_ and the Al_2_O_3_ dual‐barrier device. (c) Schematic illustration of CF configuration in the two devices. (d, e) Endurance performance (left panel) and schematic illustration of CF configuration (right panels) (d) in the single HfO_2_ device and (e) in the Al_2_O_3_ dual‐barrier device.

Meanwhile, although the insertion of baffle barriers suppressed the drift of V_O_s, it did not affect the actual switching speed. Figure 6a,b shows the experimentally obtained current responses of the single HfO_2_ device (red) and the Al_2_O_3_ dual‐barrier device (blue) to the applied voltage pulses (black lines) during the SET (+2.5 V, 0.8 µs) and RESET (−2.5 V, 0.8 µs) switching processes, respectively. During the SET switching, the switching time was 175 ns for the single HfO_2_ device and 40 ns for the Al_2_O_3_ dual‐barrier device, indicating faster switching in the latter (Figure 6a). In the single HfO_2_ device, a stronger filament can be formed in the LRS, which may result in a longer overall switching time. Taking this into account, the switching speed of the two devices can be regarded as comparable in practice. Also, the RESET switching time was similar: 80 ns for the single HfO_2_ device and 75 ns for the Al_2_O_3_ dual‐barrier device (Figure 6b).

The reason the switching speeds of the two devices are comparable is that, although the size of the residual filament is modulated by the IL, the actual switching occurs identically within the HfO_2_ region at the BE interface. Figure 6c illustrates the CF configuration that explains this behavior. In the single HfO_2_ device, V_O_ drift occurs relatively freely across the oxide layer, forming a wider CF that ruptures abruptly during RESET. In contrast, in the Al_2_O_3_ dual‐barrier device, the inserted ILs restrict V_O_ migration and enable a smaller CF formation at the BE interface. Although their CF configurations are different, the CF formation process occurs identically within the HfO_2_ region at the BE interface, and therefore the switching speed remains comparable.

Next, Figure 6d,e shows the endurance characteristics and schematic illustrations of filament evolution for the single HfO_2_ and Al_2_O_3_ dual‐barrier devices, respectively. The SET and RESET pulse amplitudes were +1.9 and −2.2 V for the single HfO_2_ device, and +2.3 and −2.1 V for the Al_2_O_3_ dual‐barrier device. The slight difference between these values arises from the distinct switching conditions of the two devices, which were optimized to achieve similar *R*_LRS_ and *R*_HRS_ levels. While the single HfO_2_ device exhibited a limited endurance of 1.6 × 10^4^ cycles, the Al_2_O_3_ dual‐barrier device sustained more than 10^6^ cycles, consistent with the simulation results in Figure 4. In the single HfO_2_ device, endurance degradation is characterized by a progressive loss of resistance modulation capability, which is observed as a gradual narrowing of the on/off resistance window during repeated cycling. Such endurance degradation behavior has been widely reported in HfO_2_‐based VCM devices and is commonly attributed to interfacial oxygen exchange and redox reactions involving V_O_s at the TE interface, which lead to vacancy depletion and reduced filament reversibility under prolonged switching stress [51, 52, 53]. Also, our experimental observation of poorer endurance in the HfO_2_‐based VCM with an oxygen‐scavenging Ti top electrode further supports the proposed endurance degradation mechanism (Figure S11). In contrast, in the Al_2_O_3_ dual‐barrier device, the IL effectively confines V_O_ migration and suppresses V_O_ accumulation, resulting in stable and durable switching behavior.

In addition, interlayer engineering has been widely reported to reduce device‐to‐device (D‐to‐D) variability by constraining stochastic filament configurations [13, 54, 55]. Consistent with this trend, dual‐barrier device exhibits improved D‐to‐D uniformity compared with the single‐layer device (Figure S12), representing an additional benefit of the multilayer architecture.

## 3 Conclusion

In this study, we proposed a memristive baffle system that controls the migration of V_O_s, achieving up to a 43% improvement in synaptic linearity characteristics and more than a 60× enhancement in endurance compared with conventional memristors from a 5‐layer dual‐barrier architecture.

This 5‐layer dual‐barrier architecture is extremely difficult to arrive at through purely combinatorial or trial‐and‐error exploration. Instead, our ability to identify and select this configuration was made possible through the introduction of a multiscale simulation technique combined with a simulation–experiment co‐design approach. The inaccuracy of material parameters, a key limitation of conventional simulations, was addressed using DFT calculations, enabling the developed simulation framework to provide reliable guidelines for exploring new materials and architectures. The 5‐layer memristor fabricated based on these simulations exhibited the expected characteristics, confirming the validity of the design strategy.

Conclusively, accurate modeling can serve as a practical guideline for device fabrication. While our framework treats V_O_s as the primary mobile species, recent reports suggest that cationic defects may also participate in switching dynamics under certain conditions. Incorporating such additional mobile species into future VCM simulation frameworks would enable a more comprehensive and refined description of defect transport and filament evolution. With continued advances along these directions, we anticipate that such simulation‐guided experimental design will become increasingly important for future materials and device development, especially when combined with emerging data‐driven and AI‐assisted tools that further accelerate predictive exploration of complex design spaces.

## 4 Experimental Section

### 4.1 Device Fabrication

Devices with the stack structure of Pt (10 nm)/TiN (40 nm)/resistive switching (RS) layer/Pt (40 nm)/Ti (5 nm) were fabricated on a SiO_2_ (300 nm)/Si substrate using the following procedure. A 5 nm‐thick Ti adhesion layer was deposited on the substrate by e‐beam evaporation. Subsequently, a 40 nm‐thick Pt BE was deposited by e‐beam evaporation. These layers were patterned through a lift‐off process. For the RS layers, the following various multilayer stacks were fabricated: 14 nm‐thick HfO_2_ in the single HfO_2_ device, HfO_2_ (2 nm)/Al_2_O_3_ (2 nm)/HfO_2_ (10 nm) for the top IL device, HfO_2_ (10 nm)/Al_2_O_3_ (2 nm)/HfO_2_ (2 nm) for the bottom IL device, and HfO_2_ (2 nm)/Al_2_O_3_ (2 nm)/HfO_2_ (6 nm)/Al_2_O_3_ (2 nm)/HfO_2_ (2 nm) for the Al_2_O_3_ dual‐barrier device. HfO_2_ films were deposited by plasma‐enhanced atomic layer deposition (PEALD) using Tris(dimethylamino)(cyclopentadienyl)hafnium (CpHf) as the Hf precursor and O_2_ plasma as the oxidant. Al_2_O_3_ films were deposited by atomic layer deposition (ALD) using Trimethylaluminum (TMA) as the Al precursor and H_2_O as the oxidant. Then, a 40 nm‐thick TiN TE was deposited by DC reactive sputtering using a TiN metal target at room temperature. A 10 nm‐thick Pt passivation layer was deposited on top of the TiN by e‐beam evaporation. These layers were patterned through a lift‐off process. The line widths of TE and BE were 5 µm.

### 4.2 DFT Calculation

The density functional theory (DFT) calculations were performed using the Vienna Ab initio Simulation Package (VASP) [56, 57]. The PBE exchange‐correlation functional was used [58] under the projected augmented wave (PAW) potential [59, 60]. For monoclinic HfO_2_, 2 × 1 × 1, 1 × 2 × 1, and 1 × 1 × 2 supercells (Hf_8_O_16_) were generated, with all the possible configurations containing 1, 2, 3, and 4 V_O_s via LACOS package [61]. The energy cutoff was set to 500 eV for the plane‐wave basis set, and the self‐consistency was met when the energy differences were below 10^−6^ eV. Gaussian smearing was applied with a smearing width of 0.01 eV. For the ionic relaxation, the cells were relaxed until the Hellmann‐Feynman forces were below 0.02 eV/Å, with the corresponding Monkhorst‐Pack k‐point meshes for each supercell being 4 × 8 × 8, 8 × 4 × 8, and 8 × 8 × 4, respectively. The electronic conductivity was calculated using the BoltzTraP2 package [62] from the band structures calculated with denser k‐point meshes (8 × 16 × 16, 16 × 8 × 16, and 16 × 16 × 8). The effective conductivity for each composition (HfO_1.875_, HfO_1.75_, HfO_1.625_, and HfO_1.5_) and temperature (300−500 K) was calculated as a weighted average, where the weight is proportional to the Boltzmann distribution of the relative energies of each configuration for a given composition.

## Conflicts of Interest

The authors declare no conflicts of interest.

## Supporting information




**Supporting File**: advs74377‐sup‐0001‐SuppMat.pdf.

## Data Availability

The data that support the findings of this study are available from the corresponding author upon reasonable request.
