# Supplementary material for: Memristive Baffle Systems: Design, Simulation, and Applications
Source: Adv Sci (Weinh). 2026 Mar 3;13(23):e23273. doi: 10.1002/advs.202523273 (PMC13104148; doi:10.1002/advs.202523273)
Supplement: Supplementary file 1 — Supporting File: advs74377‐sup‐0001‐SuppMat.pdf. [file ADVS-13-e23273-s001.pdf]

Supporting Information

Memristive Baffle Systems: Design, Simulation, and Applications

Eun Young Kim, Juseong Park, Sumin Ju, Taeyoung Jeong, Dohyun Kim, Woojoon Park, Myeongchan Ko, Woon Hyung Cheong, Jung-Hae Choi, and Kyung Min Kim**

This Supporting Information contains the following materials:

Figure S1. The geometric structure and boundary conditions of VCM memristor in the COMSOL Multiphysics simulation.
Table S1. Equations to describe the resistive switching of VCM device in multiphysics modeling.
Figure S2. DFT-based evaluation of oxygen vacancy configurations and their stability in sub-stoichiometric HfO_x.
Table S2. Compositions, supercell structures, and configurations in the DFT calculations.
Figure S3. DFT-based analysis of the effective conductivity and conductive pathways in HfO_x with varying oxygen vacancy concentrations.
Table S3. Material properties used in multiphysics modeling.
Figure S4. Effects of interlayer material parameters on device performance in simulation.
Figure S5. Comparison of V_O distributions depending on the type of interlayer material.
Figure S6. Simulation results of HfO_2-based with SiO_2 interlayers.
Table S4. Comparison of material properties for candidate interlayer materials.
Figure S7. COMSOL simulation geometries and schematic representations of V_O distribution for two memristor device structures.
Figure S8. Simulation results of the single HfO_2 device and the Al_2O_3 dual-barrier device.
Figure S9. Simulated temperature and electric field distributions along the z-direction during the reset switching.
Figure S10. Endurance characteristics of devices with a single Al_2O_3 interlayer.
Figure S11. Endurance characteristics of HfO_2-based devices with different top electrodes.
Figure S12. Device-to-device (D-to-D) variability analysis of single HfO_2 and Al_2O_3 dual-barrier devices.

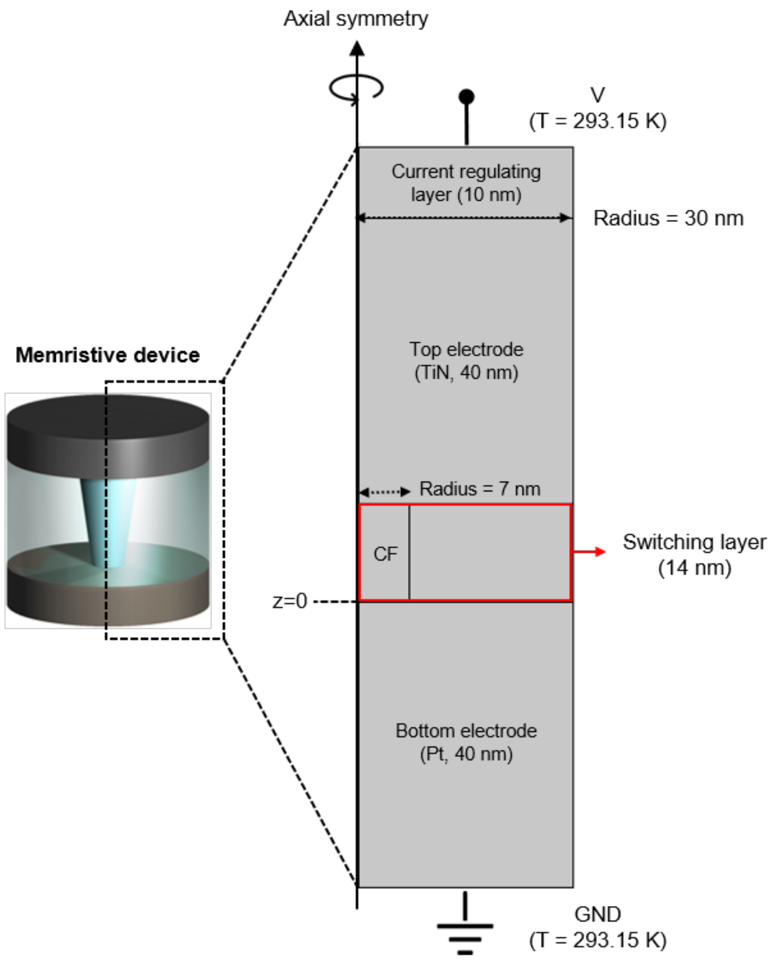

Figure S1. The geometric structure and boundary conditions of VCM memristor in the COMSOL Multiphysics simulation. The simulated geometry was designed to represent the actual device configuration. A current-regulating layer (10 nm thick) was included to emulate compliance current behavior in the simulation. When the applied voltage is V_1, and the actual voltage across the device is V_2, the conductance σ of this layer is defined as:

$$\sigma = \frac{I_{cc} \times L}{(V_1 - V_2) \times \pi r^2} \quad [1]$$

The top electrode is TiN (40 nm), and the bottom electrode is Pt (40 nm). The resistive switching layer, including the interlayer, has a total thickness of 14 nm. A conducting filament (CF), representing an oxygen vacancy-deficient region, is implemented at the center of the switching layer with a radius of 7 nm. Axial symmetry and thermal boundary conditions were applied at both electrodes with a temperature of 293.15 K.

Table S1. Equations to describe the resistive switching of VCM device in multiphysics modeling[2].

**Device physics**	**Equations**
Oxygen vacancy transport	$\frac{\partial n_D}{\partial t} = \nabla(\overline{D}\nabla n_D + \overline{D}Sn_D\nabla T - vn_D)$
Current continuity	$\nabla \cdot J_v = Q_{j,v}$
Fourier equation for Joule heating	$\rho C_P \frac{\partial T}{\partial t} + \rho C_P \mathbf{u} \cdot \nabla T = \nabla \cdot (k\nabla T) + Q_e$
**Parameters**	**Equations**
Pre-exponential factor for diffusion coefficient	$\overline{D}_0 = \frac{1}{6}\bar{z}\bar{a}^2\bar{v}$
Diffusion coefficient for random work process	$\overline{D}_{rand} = \overline{D}_0 \exp(-\overline{E}_a/k_B T)$
Diffusion coefficient for actual diffusion process	$\overline{D} = f \cdot \overline{D}_{rand}$
Drift velocity	$v = a \times f \times \exp\left(-\frac{E_a}{kT}\right) \times \sinh(\frac{qaE}{kT})$
Soret diffusion coefficient	$S = -\frac{E_a}{kT^2}$

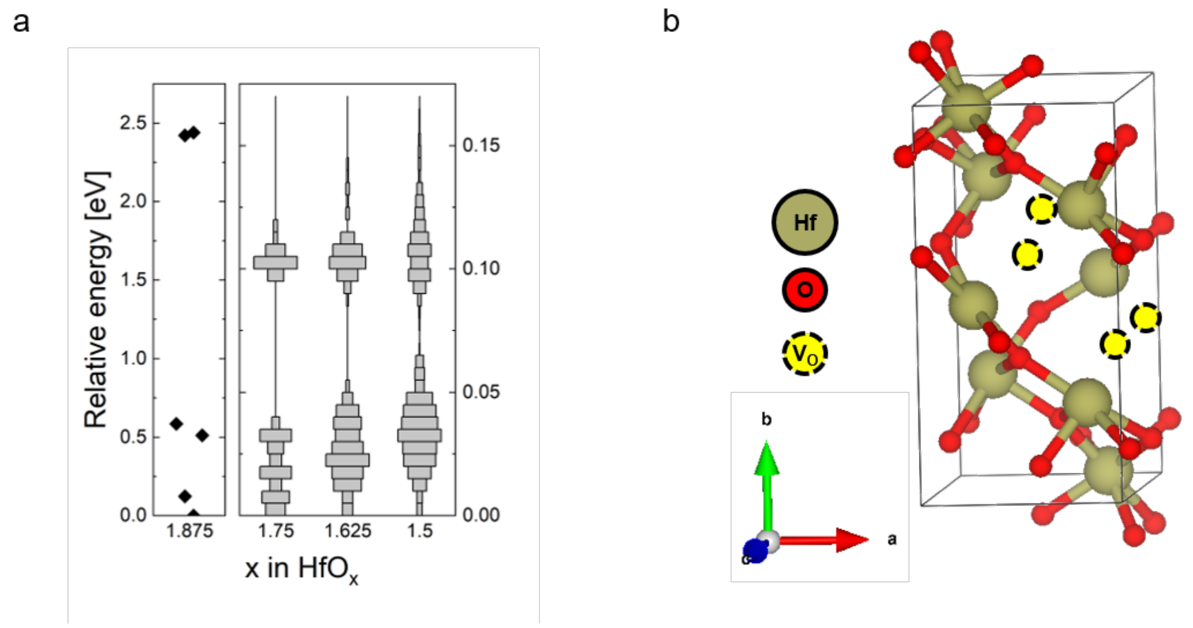

Figure S2. DFT-based evaluation of oxygen vacancy configurations and their stability in sub-stoichiometric HfO_x. (a) Energetic distribution of the various configurations in HfO_x supercells and (b) the most energetically stable configuration in $HfO_{1.5}$.

To calculate the electronic conductivity σ of the conductive filament (CF) region, the Boltzmann transport equation is applied, based on the DFT calculations for monoclinic HfO_{2-x} containing oxygen vacancies (V_Os). To model the anisotropy of oxygen-deficient monoclinic phase, the 2×1×1, 1×2×1, and 1×1×2 supercells were investigated (i.e., Hf_8O_{16-n}, n=1~4) and all the inequivalent configurations containing V_Os for each supercell were generated via the LACOS package[3]. The number of configurations in each composition and supercell investigated is summarized in **Table S2**.

The density functional theory (DFT) calculations were performed using the Vienna Ab initio Simulation Package (VASP)[4, 5]. The PBE exchange-correlation functional was used[6] under the projected augmented wave (PAW) potential[7, 8]. The energy cutoff was set to 500 eV for the plane-wave basis set, and the self-consistency was met when the energy differences were below 10^{-6} eV. Gaussian smearing was applied with a smearing width of 0.01 eV. For ionic

relaxation, the supercells were relaxed until the Hellmann-Feynman forces were below 0.02 eV/Å with the corresponding Monkhorst-Pack k-point meshes for each supercell being 4×8×8, 8×4×8, and 8×8×4, respectively.

Table S2. Compositions, supercell structures, and configurations in the DFT calculations.

**n in Hf_8O_{16-n}**	**Composition**	**Number of inequivalent configurations**		
		**2×1×1 supercell**	**1×2×1 supercell**	**1×1×2 supercell**
1	Hf_8O_{15} ($HfO_{1.875}$)	2	2	2
2	Hf_8O_{14} ($HfO_{1.75}$)	22	20	20
3	Hf_8O_{13} ($HfO_{1.625}$)	70	70	70
4	Hf_8O_{12} ($HfO_{1.5}$)	252	246	246

The σ was calculated using the BoltzTraP2 package[9] from the density of states (D(E)) obtained in DFT calculations. For the electronic structure calculations, denser k-point meshes of 8×16×16, 16×8×16, and 16×16×8 were used for the 2×1×1, 1×2×1, and 1×1×2 supercells and the σ of each configuration, θ, at each composition, *x*, was calculated using **Equation S1**. τ is the relaxation time of the scattering, which was assumed to be constant as 10^{-14} s, based on the relaxation time approximation. v is the velocity of the electron.

$$\sigma_{\theta,x} = e^2 \int D(E) \cdot \tau \cdot v(k)^2 dk \qquad \textbf{(Eq. S1)}$$

Within a given *x*, on the other hand, the probability for a θ being formed, P_θ, is proportional to the Boltzmann distribution compared to the energetically most stable configuration.

$$P_\theta \propto \exp\left(\frac{-(E_\theta - \min(E_\theta))}{k_B T}\right) \qquad \textbf{(Eq. S2)}$$

That is, the θ with higher energy is less likely to form and contributes less to the effective conductivity, σ_{eff} of that *x*. The P_θ was normalized for each *x* (i.e., HfO_x, x =1.875, 1.75, 1.625

and 1.5) and temperature (300−500 K). The effective conductivity is obtained as the weighted average, where the weight is the normalized P_θ.

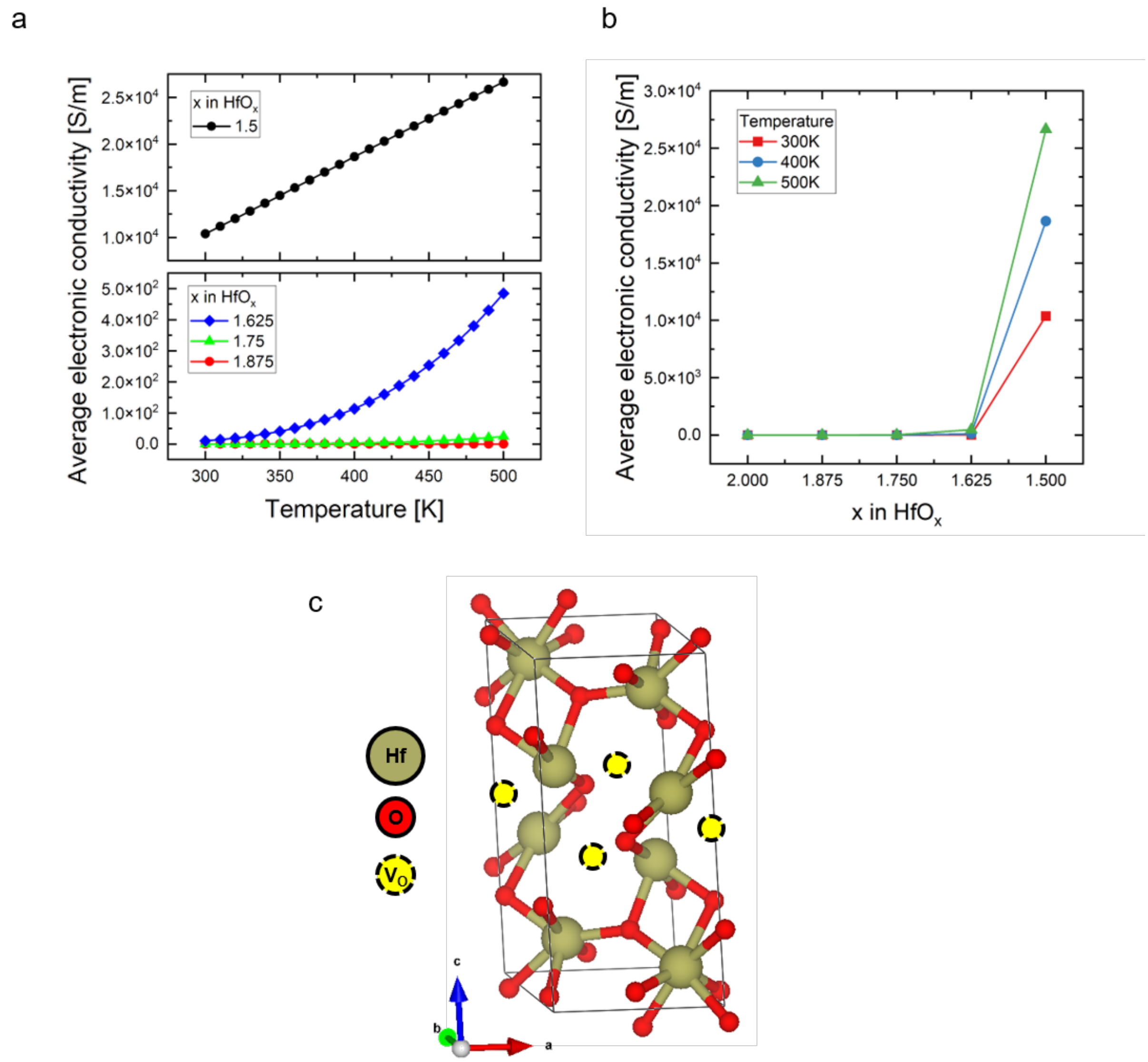

Figure S3. DFT-based analysis of the effective conductivity and conductive pathways in HfO_x with varying oxygen vacancy concentrations. (a) Calculated effective conductivity as a function of temperature. It shows the σ_{eff} of each x over a temperature range from 300 K to 500 K. For $HfO_{1.5}$, the conductivity is estimated to be 10^4 S/m at 300 K, which is at least two orders of magnitude higher than that of the other compositions. (b) Calculated effective conductivity as a function of composition for monoclinic HfO_x. (c) The configuration in $HfO_{1.5}$ with the highest conductivity at 300 K, where the V_Os are also clustered and form a conducting path in the a-b plane.

Table S3. Diffusion parameters of monoclinic HfO_2[10].

Effective diffusion parameter (monoclinic HfO_2)		
$\bar{D}_0$	2.86×10^{-7} m^2/s	pre-exponential for diffusion coefficient
$\bar{E}_a$	0.70 eV	diffusion barrier
f	0.32	correlation factor
$\bar{\nu}$	5.24 THz	jump attempt frequency
$\bar{a}$	2.67 Å	hopping distance
$\bar{z}$	4.58	coordination number

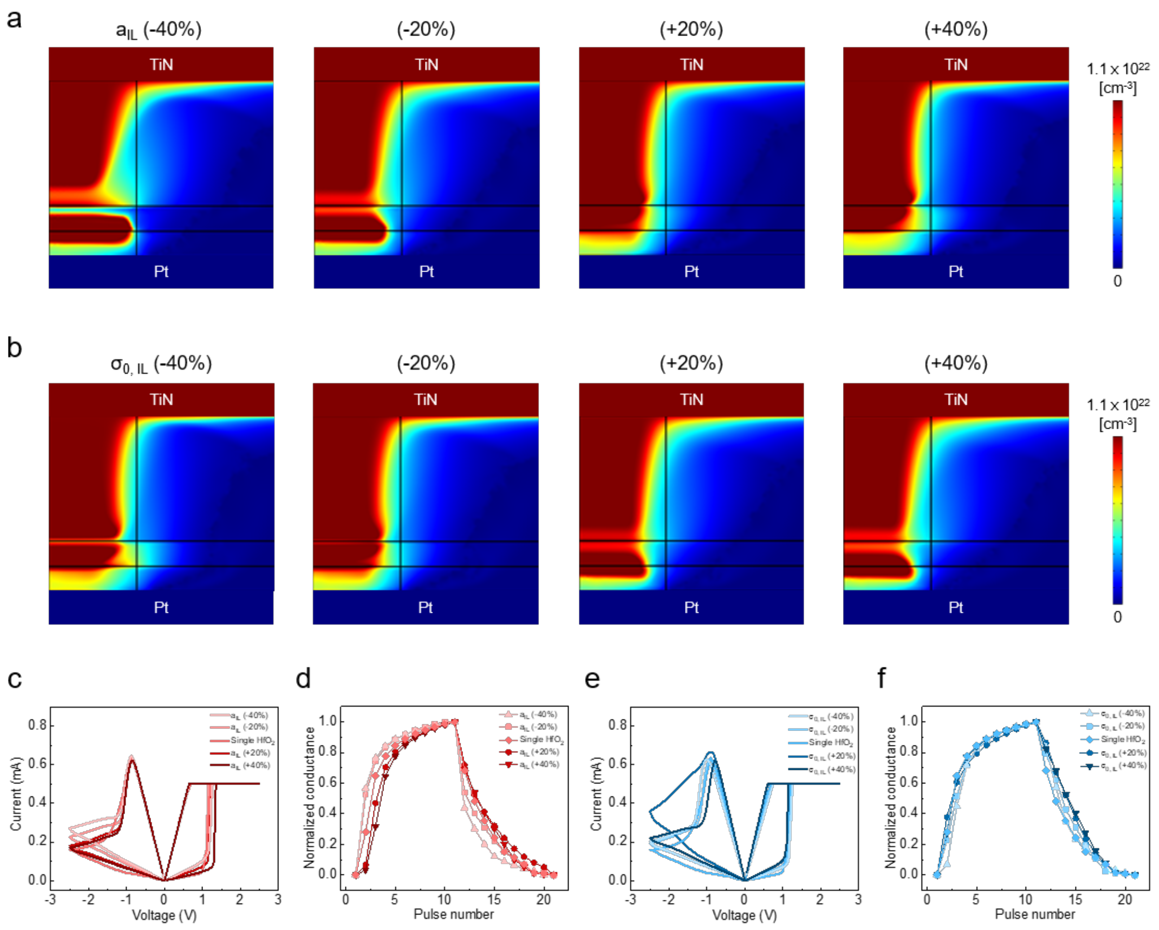

Figure S4. Effects of interlayer material parameters on device performance in simulation. (a) V_O concentration distributions for devices with varying hopping distance in the interlayer (a_{IL}). (b) V_O concentration distributions for devices with various pre-exponential factors of electrical conductivity in the interlayer ($\sigma_{0,\ IL}$). (c) *I-V* characteristics for devices by variations in a_{IL}. (d) Potentiation and depression behavior under pulse application, showing how synaptic linearity and conductance modulation are influenced by variations in a_{IL}. (e) *I-V* curve comparison for devices by variations in $\sigma_{0,\ IL}$. (f) Potentiation and depression behaviors by variations in $\sigma_{0,\ IL}$.

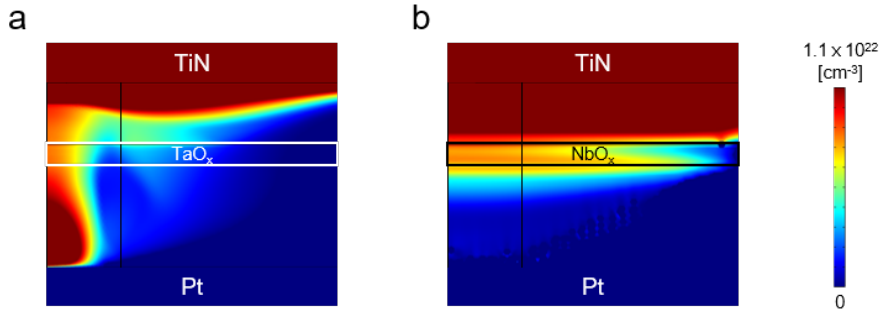

Figure S5. Comparison of V_O distributions depending on the type of interlayer material. (a) V_O distribution in the HfO_2 device with a 2 nm Ta_2O_5 interlayer. The model exhibits enhanced penetration of V_O, indicating facilitated migration pathways across the switching layer. (b) V_O distribution in the HfO_2 device with a 2 nm Nb_2O_5 interlayer. The model shows a broader dispersion of V_O around the interlayer. The distributions shown were obtained after the set switching process.

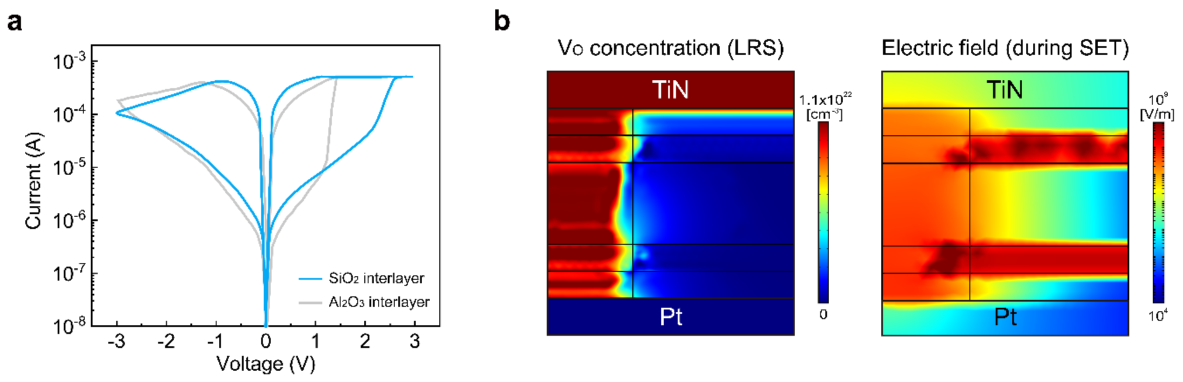

Figure S6. Simulation results of HfO_2-based devices with SiO_2 interlayers. (a) Simulated *I-V* curve comparing dual SiO_2 interlayer and dual Al_2O_3 interlayer devices. (b) Simulated V_O distribution (left) and electric field distribution in the device with dual SiO_2 interlayers (right).

Table S4. Comparison of material properties for candidate interlayer materials.

	Activation energy (E_a)	Dielectric constants (ε)	Crystallization temperature	Oxygen vacancy formation energy (E_{form})
Al_2O_3	0.9 eV[11]	8 - 9	800 °C	4.0 – 7.0 eV[12]
Ta_2O_5	0.4 - 1.1 eV[13]	25	650 °C	4.4 – 6.3 eV[14]
SiO_2	2.1 - 2.2 eV[15]	3.9	1000 °C	8 eV[16]
Nb_2O_5	0.7 – 1.0 eV[17]	28	550 °C	6 eV[18]

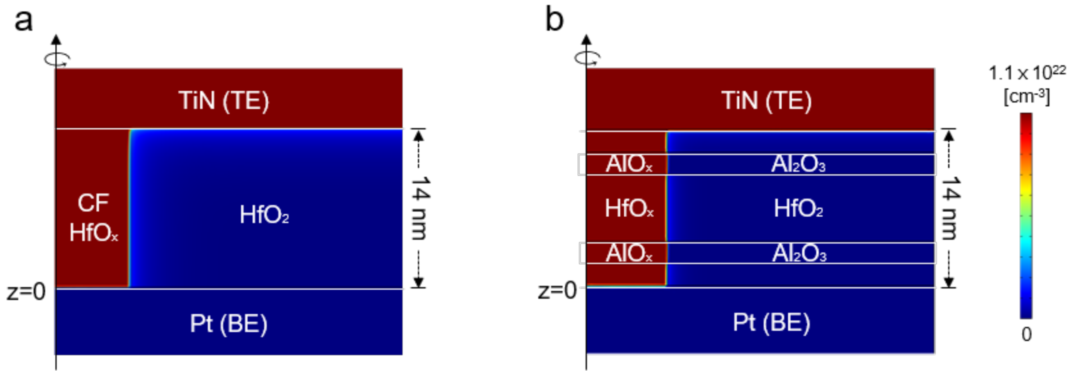

Figure S7. COMSOL simulation geometries and schematic representations of V_O distribution for two memristor device structures. (a) A reference memristor structure consisting of a TiN/HfO_2/Pt stack. A localized oxygen-deficient region (HfO_x) containing a conductive filament (CF) is included to model resistive switching behavior. (b) Simulation geometry of a device with Al_2O_3 dual interlayers, where AlO_x bilayers are inserted within the HfO_2 switching layer to modulate the oxygen vacancy profile and filament evolution. Both models use a total switching layer thickness of 14 nm.

To analyze the impact of Al_2O_3 layers on bulk HfO_2, we conducted device modeling and simulations. **Figure S7a** shows the structure of the single HfO_2 device used in the simulations, while **Figure S7b** shows the structure of the Al_2O_3 dual-barrier device. Both models were based on a 2D symmetrical configuration. The device stack, from top to bottom, consisted of the following layers: a current modulation layer[19] (10 nm), a top electrode (TiN, 30 nm), a resistive switching layer (14 nm), and a bottom electrode (Pt, 30 nm). In the single HfO_2 device, the resistive switching layer consisted solely of HfO_2 (14 nm). In contrast, the Al_2O_3 dual-barrier device featured a multilayer configuration of HfO_2 (2 nm)/Al_2O_3 (2 nm)/HfO_2 (6 nm)/Al_2O_3 (2 nm)/HfO_2 (2 nm).

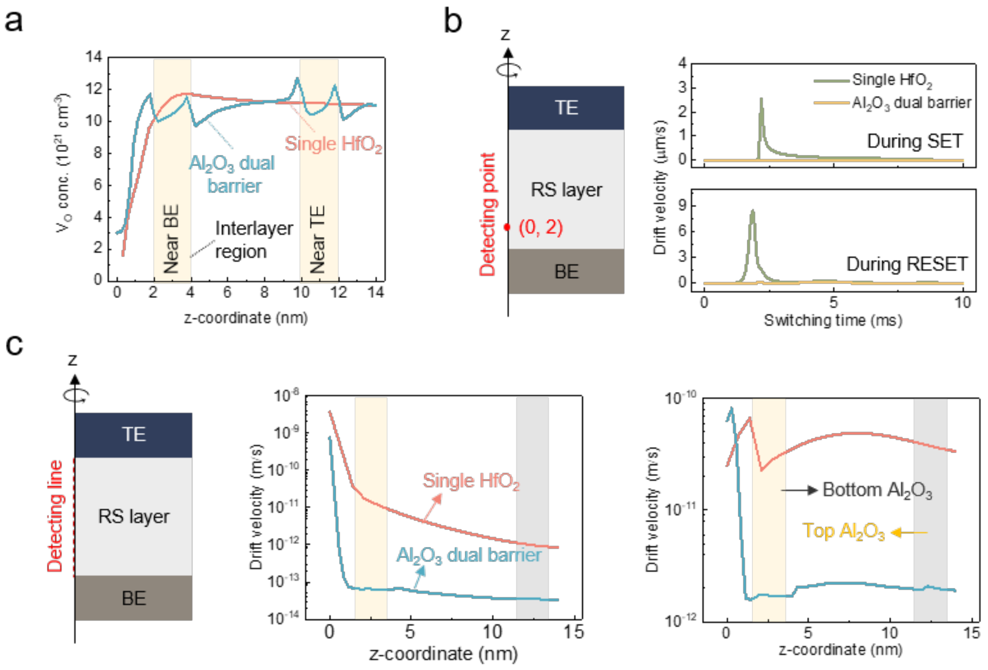

Figure S8. Simulation results of the single HfO_2 device and the Al_2O_3 dual-barrier device. (a) Depth profiles of the V_O concentration after SET in both devices, highlighting the effect of the Al_2O_3 interlayer regions. (b) (left) Schematic of the detecting point, and (right) simulated v_d values during SET (top) and RESET (bottom). (c) (left) Schematic illustration of the detecting line across the switching layer. (middle) Simulated v_d profiles along the detecting line during SET. (right) Simulated v_d profiles during RESET for each device structure.

Figure S8a plots the V_O concentration over the z-coordinate after SET switching for both device models. In the Al_2O_3 IL regions (2 nm < z < 4 nm and 10 nm < z < 12 nm), V_O concentrations were lower compared to the HfO_2 regions, with V_O accumulation observed at the HfO_2/Al_2O_3 interfaces.

To explore the relationship between the kinetic mechanisms involved in the formation and rupture of CF and their impact on linearity, simulations were conducted to measure and compare the drift velocity (v_d) of V_O at specific locations within the CF. **Figure S8b, left** illustrates the detecting point (0, 2), which are used to trace v_d values during both set and reset switching over time. In **Figure S8b, right**, the single HfO_2 device consistently exhibited higher

v_d values at both points, especially during the initial stages of voltage application. This trend emphasizes the role of the Al_2O_3 IL, which, due to its high V_O migration energy, maintains lower v_d values throughout the switching process.

Figure S8c, left presents a schematic illustration of the detecting line across the switching layer under maximum set and reset voltages. During set switching (**Figure S8c, middle**), the v_d of the single HfO_2 device (red line) was generally higher than that of the Al_2O_3 dual-barrier device (blue line). A similar trend was observed throughout the CF region during reset switching (**Figure S8c, right**), where the single HfO_2 device displayed significantly higher v_d values, particularly close to the bottom electrode (BE). Here, the v_d increased due to the strong electric field. However, in the region near the BE ($z < 0.57$ nm), the v_d of the Al_2O_3 dual-barrier device was slightly higher. This behavior is attributed to the stronger electric field generated within the Al_2O_3 layer, which has a relatively high migration barrier energy[5].

Conversely, while the electric field in the HfO_2 layer was weaker, the Al_2O_3 layer focused the field, transmitting it to the adjacent HfO_2 region. This combination of a stronger electric field and a lower energy barrier in the HfO_2 layer resulted in areas where v_d was higher than in the single HfO_2 device.

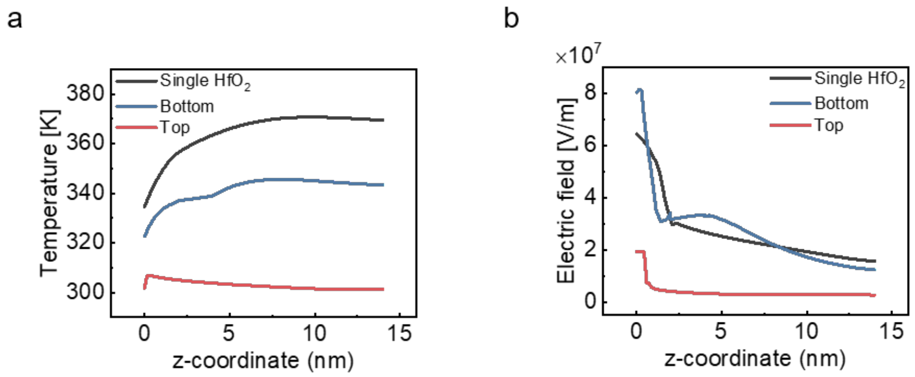

Figure S9. Simulated (a) temperature and (b) electric field distributions along the z-direction during the reset switching. Among the models, the top interlayer model (red curve) exhibits the most uniform distribution in both temperature and electric field, compared to the single HfO_2 model (gray curve) and the bottom interlayer model (blue curve).

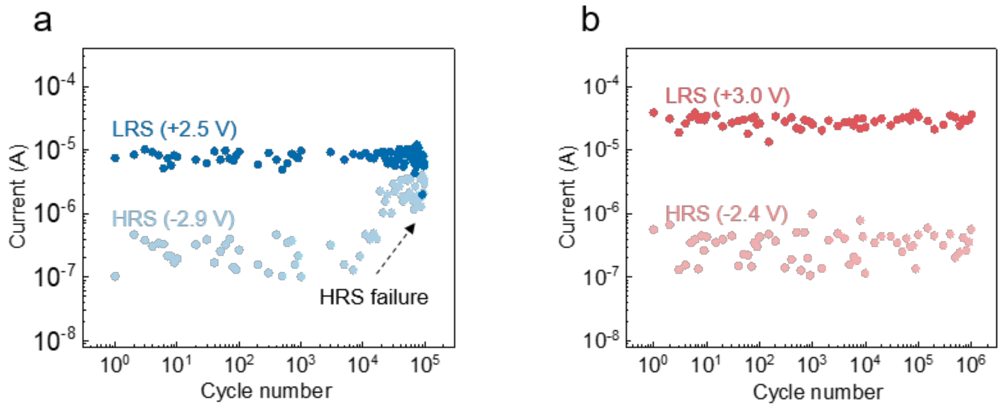

Figure S10. Endurance characteristics of devices with a single Al_2O_3 interlayer. (a) Single bottom Al_2O_3 interlayer device, showing HRS degradation and failure after 10^4 cycles. and (b) Single top Al_2O_3 interlayer device, which maintains stable resistance states up to 10^6 cycles without abrupt failure.

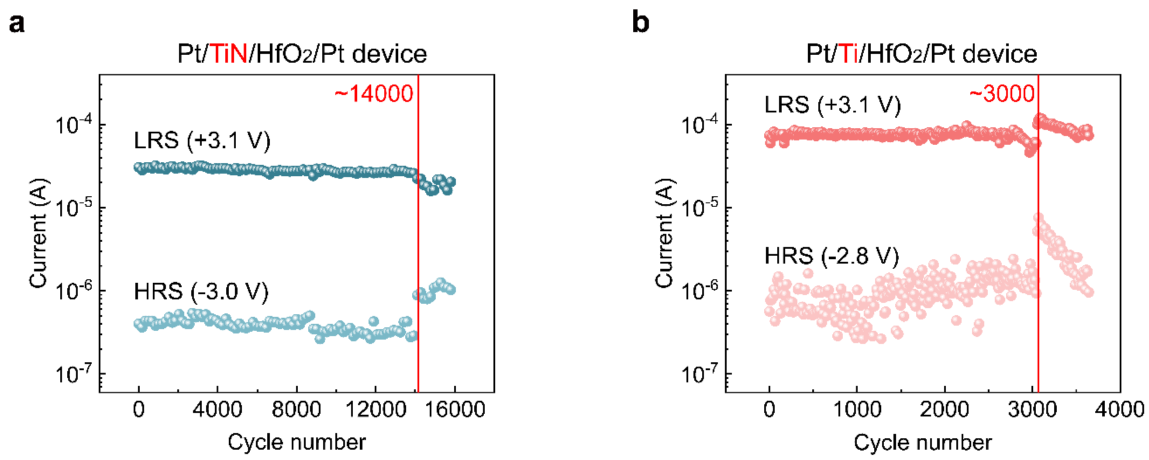

Figure S11. Endurance characteristics of HfO_2-based devices with different top electrodes. Endurance characteristics of (a) Pt/TiN/HfO_2/Pt and (b) Pt/Ti/HfO_2/Pt devices measured under pulsed switching conditions. The device with a TiN top electrode exhibits stable LRS and HRS characteristics over approximately 1.4×10^4 switching cycles, whereas the device with a Ti top electrode shows a pronounced degradation of resistance states after approximately 3×10^3 cycles. The reduced endurance observed in the Ti top electrode device is attributed to the higher oxygen affinity of Ti, which promotes interfacial oxygen exchange and accelerates degradation during repeated switching.

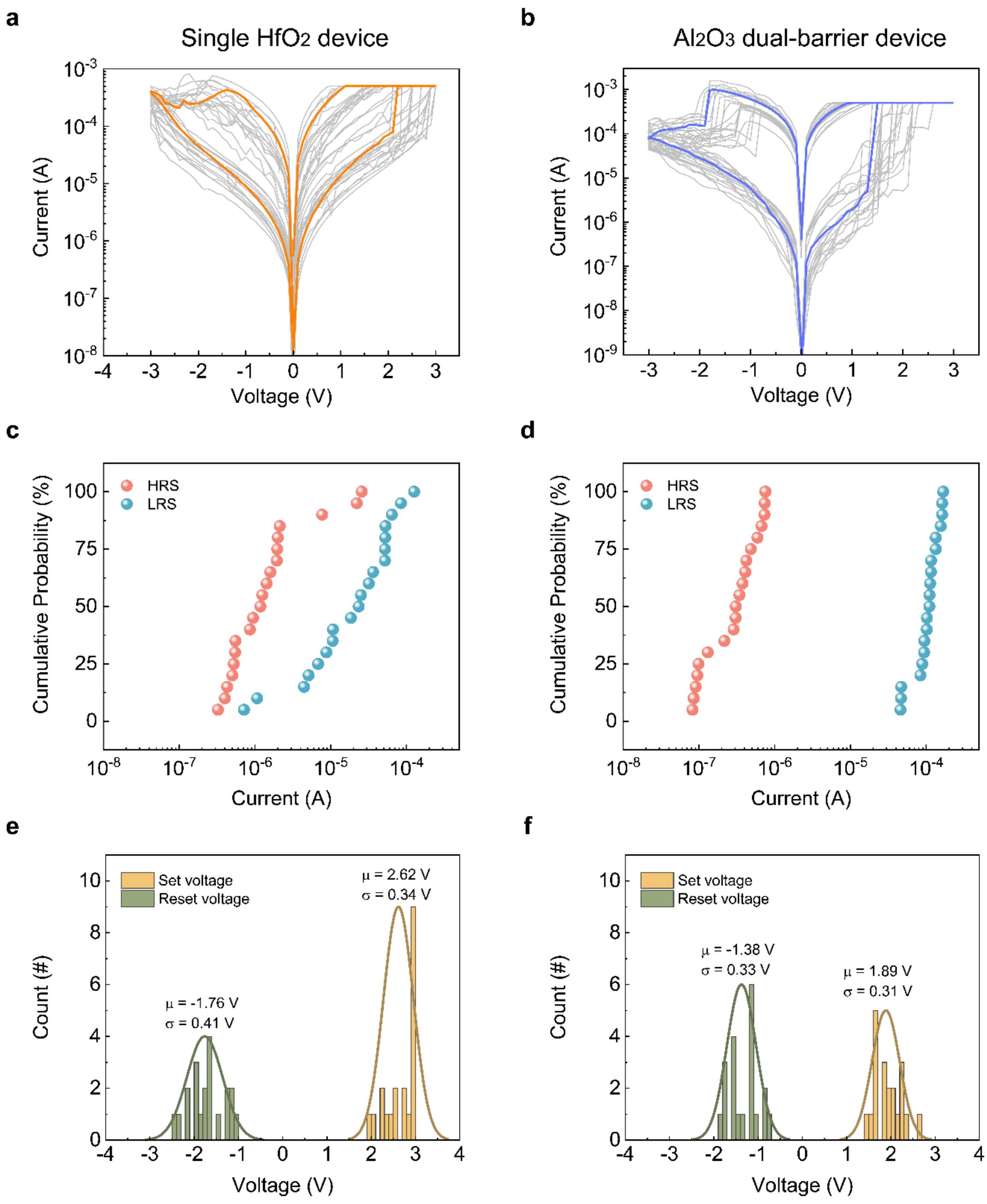

Figure S12. Device-to-device (D-to-D) variability analysis of single HfO_2 and Al_2O_3 dual-barrier devices. Overlaid DC *I-V* characteristics measured from multiple devices fabricated under identical process conditions for the (a) single HfO_2 device and (b) Al_2O_3 dual-barrier device, showing D-to-D dispersion in switching behavior. Cumulative probability distributions of HRS and LRS currents extracted from different devices for the (c) single HfO_2 device and (d) Al_2O_3 dual-barrier device. The dual-barrier device exhibits narrower and more consistently

separated HRS and LRS distributions. Statistical distributions of set and reset voltages for the (e) single HfO_2 device and (f) Al_2O_3 dual-barrier device. Solid lines represent Gaussian fits, from which the mean (μ) and standard deviation (σ) are extracted to quantify D-to-D variability.

[1] J. Park, J. Choi, G. Kim, G. Kim, G.S. Kim, H. Song, Y.S. Kim, Y. Lee, H. Rhee, H.M. Lee, C.S. Hwang, and K.M. Kim, "Modified Dynamic Physical Model of Valence Change Mechanism Memristors", *ACS Appl Mater Interfaces*, **2022**. 14(31): p. 35949-35958.

[2] S. Kim, S. Choi, and W. Lu, "Comprehensive physical model of dynamic resistive switching in an oxide memristor", *ACS Nano*, **2014**. 8(3): p. 2369-2376.

[3] M. Chandran, "Multiscale ab initio simulation of Ni-based alloys: Real-space distribution of atoms in γ+ γ′ phase", *Computational Materials Science*, **2015**. 108: p. 192-204.

[4] G. Kresse and J. Furthmüller, "Efficient iterative schemes for ab initio total-energy calculations using a plane-wave basis set", *Physical review B*, **1996**. 54(16): p. 11169.

[5] G. Kresse and J. Furthmüller, "Efficiency of ab-initio total energy calculations for metals and semiconductors using a plane-wave basis set", *Computational materials science*, **1996**. 6(1): p. 15-50.

[6] J.P. Perdew, K. Burke, and M. Ernzerhof, "Generalized gradient approximation made simple", *Phys. Rev. Lett.*, **1996**. 77(18): p. 3865.

[7] P.E. Blöchl, "Projector augmented-wave method", *Physical review B*, **1994**. 50(24): p. 17953.

[8] G. Kresse and D. Joubert, "From ultrasoft pseudopotentials to the projector augmented-wave method", *Physical review b*, **1999**. 59(3): p. 1758.

[9] G.K. Madsen, J. Carrete, and M.J. Verstraete, "BoltzTraP2, a program for interpolating band structures and calculating semi-classical transport coefficients", *Comput. Phys. Commun.*, **2018**. 231: p. 140-145.

[10] T. Jeong, K.H. Ye, S. Yoon, D. Kim, Y. Kim, and J.-H. Choi, "VacHopPy: A Python package for vacancy hopping analysis based on ab initio molecular dynamics simulations", *arXiv preprint arXiv:2503.23467*, **2025**.

[11] L. Goux, K. Sankaran, G. Kar, N. Jossart, K. Opsomer, R. Degraeve, G. Pourtois, G.-M. Rignanese, C. Detavernier, and S. Clima. in *2012 Symposium on VLSI Technology (VLSIT)*. 2012. IEEE.

[12] D. Liu, S. Clark, and J. Robertson, "Oxygen vacancy levels and electron transport in Al2O3", *Appl. Phys. Lett.*, **2010**. 96(3).

[13] M.S. Islam, S. Ganguli, J.J. Yang, and A.K. Roy, "Diffusion Characteristics of Ru and Oxygen Vacancies in Ta2O5 for Resistive Random Access Memory Devices: A Density

Functional Theory Investigation", *Advanced Electronic Materials*: p. 2500128.

[14] Y. Guo and J. Robertson, "Oxygen vacancy defects in Ta2O5 showing long-range atomic re-arrangements", *Appl. Phys. Lett.*, **2014**. 104(11).

[15] L.R. Corrales, J. Song, R. VanGinhoven, and H. Jonsson, "A Comparative Study of Oxygen Vacancy Migration Pathways in Crystalline Polymorphs of Silica", *Ceram. Trans.*, **2000**. 107: p. 139-150.

[16] D.Z. Gao, J. Strand, M.S. Munde, and A.L. Shluger, "Mechanisms of oxygen vacancy aggregation in SiO2 and HfO2", *Frontiers in Physics*, **2019**. 7: p. 43.

[17] J. Xu, Y. Yang, X. Xu, H. Wang, Y. Zhu, Y. Liu, R. Xiong, and M. Chen, "Tailoring the Resistive Switching Performance of Niobium Oxide Memristors for Neuromorphic Computing through Nitrogen Doping", *J. Mater. Res. Technol-JMRT*, **2025**.

[18] B. Baldassarri, J. He, A. Gopakumar, S. Griesemer, A.J. Salgado-Casanova, T.-C. Liu, S.B. Torrisi, and C. Wolverton, "Oxygen vacancy formation energy in metal oxides: High-throughput computational studies and machine-learning predictions", *Chem. Mater.*, **2023**. 35(24): p. 10619-10634.

[19] J. Park, J. Choi, G. Kim, G. Kim, G.S. Kim, H. Song, Y.S. Kim, Y. Lee, H. Rhee, and H.M. Lee, "Modified dynamic physical model of valence change mechanism memristors", *ACS Appl. Mater. Interfaces*, **2022**. 14(31): p. 35949-35958.
